# Book Reviews

**Published:** 1825-03

**Authors:** 


### CRITICAL ANALYSIS
OF
### ENGLISH AND FOREIGN LITERATURE,
RELATIVE TO THE VARIOUS BRANCHES OF
### Medical Science.

Quæ laudanda forent, et quæ culpanda, vicissim

Illa, prius, cretâ; mox hæc, carbone, *notamus*.—PERSIUS.

### DIVISION I.

### ENGLISH.

ART. I.—*An Essay on Curvatures and Diseases of the Spine, including all the Forms of Spinal Distortion; to which the Fothergillian Gold Medal was awarded by the Medical Society of London, and presented, at a special General Meeting, on the 3d of May*, 1824; *with some Additions.* By R. W. BAMPFIELD, Esq. one of the Surgeons to the Royal Metropolitan Infirmary for Children; Fellow of the Medical Society of London; Author of "an Essay on Hemeralopia, or Night-Blindness," of "Practical Treatises on Tropical and Scorbutic Dysentery," &c.—Longman and Co. London, 1824. 8vo. pp. 387.

THOSE who are in the habit of perusing this Journal must be familiar with the name of Mr. BAMPFIELD, in connexion with the subject before us, since what may be called the rudiments of the present work made their appearance in three successive Numbers of this publication, about two years ago. In those papers, Mr. Bampfield explained some of the views of the nature and mode of treating spinal diseases, which are peculiarly his own. The volume before us is a connected and extended Essay on the subject, to which the Fothergillian gold medal of the Medical Society, for the year 1823, was adjudged. Our author, in his Preface, informs us—and it is right that it should be borne in mind—that this Essay was delivered to the registrar of the Society prior to the appearance of the works of Mr. SHAW, Dr. DODS, and Dr. JARROLD, to Mr. C. BELL's Lecture, or Dr. OLLIVIER's Essay on the Spinal Marrow; and therefore he hopes that he shall not be accused of plagiarism or want of originality, from any coincidence of new opinions that may be observed between this Essay and the recent works alluded to above.

Our intention in presenting to our readers an account of this work, is to abstain as much as possible from criticism,—and to give as extended an analysis of our author's opinions and doctrines as our limits will permit; a plan which, we conceive, must be more satisfactory than merely obtruding our own opinions upon subjects, many of which are still far from being universally acquiesced in.

Of the volume itself, nine chapters are devoted to the consideration of diseases of the spine, and the remaining five chapters treat of accidents, such as fractures and dislocations of the vertebræ, &c.; of spina bifida, inflammation of the medulla spinalis, or its membranes, &c. There is also an Appendix, containing the details of several cases of spinal disease.

After a few preliminary anatomical remarks on the admirable construction of the spinal column, Mr. Bampfield proceeds to give us his division of diseases of the spine, of which he treats in the following order:—1st. Curvatures and distortions of the spine; 2d. Fractures, concussions, and dislocations of the vertebræ; 3d. Spina bifida, and some other congenital defects of spinal conformation; 4th. Inflammation of the medulla spinalis, and its membranes; 5th. Of some other affections of the spine, and some diseases which are said to originate in its derangement.

We shall pass by our author's observations on the defective arrangement of this class of diseases, in the various nosological systems of CULLEN, VOGEL, SAUVAGES, and others, and proceed to say, that he proposes to divide the distortions and projections of the spine into two kinds; one of which he calls the curvature of the spine, the other the angular projection. Of the curvature there are three varieties; one of which may be called excurvation, or the curvature outwards; the other incurvation, or the curvature inwards; the third, the lateral curvature, a term which sufficiently explains itself. The angular projection has not any varieties.

We conceive it to be unnecessary to explain to our readers the meaning of the above terms; they are sufficiently obvious. In a general way it may be observed, that, though curvatures may take place in any part of the spine, they more commonly occur in the dorsal vertebræ, and that they generally take place during the growth of the body. They are met with, however, occasionally later in life; and Mr. B. mentions the case of a female labouring under an excurvation of the spine, who was forty-six years of age.

Chapter II.—We now have arrived at chapter the second, treating of *excurvation of the spine*, and which is more frequently to be met with than any other, according to our author, whose vivid and accurate description of the progress of the symptoms we cannot do better than copy:—

"The commencement of excurvation of the spine is sometimes marked by pain of the vertebræ about to be involved in the curvature, which is more particularly felt on using exercise in the erect posture, or may be, at all times, excited by pressure on the spinous processes: in the latter case, an adult would shrink from the pain, and mention it; a child would cry. Should inflammation of any of the constituent parts of the vertebral joints exist, the pain would be of a more permanent nature or of more frequent recurrence, and the patient would be considerably relieved by assuming the horizontal posture, and in a less degree by bending the spine backwards. The majority of cases are not preceded by any pain of the vertebræ, that attracts so much attention, or occasions so much suffering, as to lead to voluntary complaint. In some cases, chiefly in those accompanied with inflammation, if a sponge wrung out of hot water be passed along the spine, it will create increase of heat and pain in the part disposed to curvature; but this is not a result obtainable in the generality of cases. An adult generally states the pain to be of an obtuse kind, and feeling like a weight; but it can only be inferred that a young child feels pain, by its crying when made to sit up or walk about. At this period, if the spine be accurately examined, particularly if it be bent a little forwards, some of the spinous processes of the dorsal vertebræ, which are the seat of pain, may be observed to project more irregularly than the rest, so that they stretch the skin over them, and make it appear white and shining, resembling the phenomenon of the stretched skin over the knuckles of a clenched fist: yet this appearance of the curvature at its commencement is only of a temporary nature, as, by laying the patient in a proper horizontal posture, and using mechanical aid, the vertebral column can be restored to its spinal line. About the time the above evidence of incipient curvature is manifest, many adults will complain of a sense of weakness in their back, of a lassitude that disinclines them from exertion, and of fatigue being soon induced by exercise; all of which dispose them to seek repose and avoid motion. A child, if he have not previously gained the use of his legs, has the ability of using the lower limbs deferred long beyond the usual period children should walk; and, if he had acquired the power of walking, he will gradually lose that remarkable spirit of activity and endless motion peculiar to children, and become listless and languid, seeking its mother's lap for a resting place. Should these symptoms of weakness and indisposition to exercise lead to the frequent adoption of the recumbent posture, the curvature will increase very slowly, or its progress will be arrested for a time. Should, however, the patient not lie down more than usual, but be seated in a chair during the day, where the vertebræ will have to bear the superincumbent weight of the head, &c., especially if he walk about as much as he is able, the curvature will advance with considerable rapidity; and, after a little increase, if the patient be placed on his feet, he cannot maintain the vertebral column in the erect attitude, but inclines the upper part forwards; and he moves with a slow, hesitating, tottering gait, and is soon tired; or he cannot direct his steps to the proper point, and he stumbles, trips or crosses his legs, and falls down crying. In this state a child dreads to be put down, and when on his feet will, with tears, cling to his mother or nurse for support; and, if deprived of it, he will totter, his knees will bend, and he will soon fall, unless caught. The lower extremities gradually diminish in size as the curvature advances, and frequently lose part of their natural sensibility and temperature; the muscles exchange their plump and firm feel for a softer one, and their power daily decreases. The patient will often sit with his legs crossed; and in bed will sometimes experience pain in his knees, and convulsive actions of the muscles of the thighs, which are more particularly felt and described by the adult, whose loss of muscular power is more generally accompanied with a sense of coldness, and diminished sensibility and firmness of the extremities. If a palsy of the lower limbs be now induced, there is, of course, an end to locomotion; the muscles and cellular texture of the extremities waste and wither; the limbs become flaccid and cold, and, if lifted up, will fall down like a mass of inanimate matter; the joints are more rigid than in ordinary paralysis, and the skin frequently retains some power of sensation. This calamitous effect or symptom, however, only supervenes occasionally; for the majority of adults and minors retain sufficient muscular power of the extremities to enable them to move about in their room, with the artificial aid they can derive from the chairs, tables, and other supports that are available in their route. Should they attempt it without such extrinsic aid, or the use of crutches, or the support of friends, they can only walk a short time with such a degree of bent posture as the curvature necessarily occasions, and suddenly lean forwards and support themselves, by placing their hands above their knees, and the vertebral column then becomes curved to a great extent.

"Most patients, if not prevented by paralysis, or restrained by medical advice, retain the disposition, which they indulge in, to sit up and walk about a little through the whole progress of the disease; but where *scrofulous caries* is the cause of the curvature, the inclination to move about very frequently forsakes them, and they sit in a chair, listless, languid, often asthmatic or afflicted with dyspnœa, and endeavour to take off the superincumbent weight of the head, by placing the elbows on the table before them, or on the arms of their chair, and lodging their lower jaw on the thumbs (as on a crutch) extended across it, with the fingers directed up to the sides of the face or ears. If the patient be confined to the horizontal posture, more especially the facial, when the excurvation is in the incipient stage, no permanent or perpetual curvature will ensue, provided its use be persevered in until the dorsal muscles have recovered their energy, or the disposition to inflammation and scrofulous action of the vertebræ be removed. Should the patient incautiously continue to sit up and walk about, a curvature of the spine ensues, and gradually increases. The first appearance of projection has been described, and, in a short time after, one, two, or three spinous processes of the dorsal vertebræ may be observed to project beyond the spinal line a quarter or half of an inch: after this, the spinous processes of the vertebræ above and below those previously projecting successively protrude, until many or the whole of the dorsal vertebræ form a curve, and are projecting backwards beyond the spinal line, from an inch to even two inches, the chord line of which does not admeasure much more than one half of the length the vertebræ, of their natural dimensions and breath, would occupy. The lower cervical vertebræ

naturally incline forward, from their junction with the first dorsal; but, in the state of dorsal curvature just described, they become placed almost horizontally in the erect attitude of the body. As the curvature increases, the spinous processes of the dorsal vertebræ gradually separate from each other; but the greatest degree of separation takes place about the middle of the curve, where the space of half an inch is left between them, and the fore-finger can be easily inserted. When they are separated at this distance, it may be always inferred, with much probability, if not certainty, that the horizontal surface of the bodies of the vertebræ, of which these spinous processes are corresponding parts, are either greatly or wholly absorbed, as well as the intervertebral substances between them. Excurvation of the spine is generally accompanied with distortion of the costæ and sternum. During the formation of an excurvation, the general health becomes impaired, and particular organs and functions suffer derangement, the symptomatology of which cannot be very briefly disposed of; for, although the description drawn may enable the surgeon to distinguish the disease in its different stages, yet it is necessary to a due comprehension of it, to enumerate and examine the evils it brings in its train, and to take a review of many of the morbid effects it produces on the various structures, systems, organs, and functions of the body." (P. 9—14.)

This long quotation renders it unnecessary for us to dwell upon the affections of the muscles, which occupy several pages of the second section, since a consideration of the symptoms above enumerated will sufficiently explain the causes of their loss of power, arising, in the case of the abdominal muscles especially, from the altered position of the body, in consequence of the excurvation, by which the attachments of those muscles are brought nearer to each other.

It will be necessary to consider a little more at length the affections of the bones of the spine and chest produced by this disease; first, as relative to their distortion, and then to the disorganisation, or destruction, that ensues. With respect to the first of these effects, our author observes:—

"Taking the most simple case, in the temporary or early stages of excurvation, and when debility of the dorsal muscles is the remote cause, it sometimes happens that there is not any existing disproportion of the bony parts of the vertebral column, and the distortion is only observable in the erect or sitting posture, and the convexity of the spine is no greater than the natural motion allows, and would not be a disease, if the natural powers could permanently remove it. In this instance, the dorsal muscles are unable to counteract the gravity of the head and vertebræ, and support them erect; so that, instead of the spine maintaining a perpendicular attitude, it is bent by the superincumbent weight into a curve, whose convex side is posterior, and concave anterior; and the most projecting convexity of the curve will be formed by the vertebræ situated in the middle of the spinal pyramid. An explanation will be first attempted of the manner by which the superincumbent weight necessarily occasions the curve to project posteriorly; and, secondly, by what cause the excurvation generally takes place in the middle of the vertebral column. The first is chiefly attributable to its shape or form, the second is effected by a common law of mechanics." (P. 22, 23.)

Both these points are illustrated at considerable length, and in a manner to us very satisfactory, and with great perspicuity; but not admitting of abridgment.

The destruction of the vertebræ is accomplished by two different modes of action: one mode is the induction of caries, the other is by progressive absorption; the intervertebral substance is also destroyed by both these means. Caries of the bodies of the vertebræ is the result of slow scrofulous inflammation, which peculiarly invades their cancellous structure, and which is connected with a soft condition of the remaining portions of the bodies of the vertebræ. Our author notices the irregularity with which the process of destruction is sometimes carried on; and observes, that inflammation of the soft parts, ending in collections of pus, bursting either externally or in the thorax, making their way sometimes to the anus or into the theca vertebralis, &c. frequently occur. The destruction of the bodies of the vertebræ by progressive absorption, commences always on their horizontal surfaces, proceeding obliquely from the anterior to the posterior part; so that the bodies of the vertebræ are first reduced to a cuneiform shape, and are then rendered thinner and thinner, until they are wholly absorbed; and the intervertebral cartilage becomes absorbed at the same time. No abscesses form in these cases.

The form and extent of the curvature will, of course, depend upon the number of vertebræ affected by either variety of the disease; and our author gives some useful hints to guide the judgment of the practitioner on this point. He also remarks, that, though it is usual to say that the bodies of the vertebræ are *entirely* destroyed, still, by a wise provision of nature, the posterior parts of those bodies, which defend the anterior portion of the spinal canal, are always the last to yield, and seem to possess a stronger power of resistance than those parts which have no such important purpose to fulfil; though, from the fatal and sudden result of a case under our author's care, where the patient (seven years of age) accidentally fell from her nurse's arms, whilst removing from her bed, and died almost immediately, he is induced to believe that in this condition of the spine fracture is very easily produced.

The derangement of the form and structure of the ribs is next noticed, and the symptoms consequent thereupon.

The three following sections are devoted to the consideration of the affections of the spinal canal, the medulla spinalis and nerves, to the condition of the ligaments, and the arterial and absorbent systems, under this form of spinal disease, and are each treated of at some length: and, if we do not pause to extract any portion of these pages, it is merely because they do not present any thing that strikes us as novel. The observations of our author are characterised by his usual good sense, and show that there is no part of his subject which he has not examined with minuteness and attention. We shall find, further on in the volume, some remarks upon relaxation of the ligaments of the spine, which demand our attention.

Section 7th.—It will easily be understood that, when the distortion of the spine is considerable, the relative situations of some or all of the viscera of the thorax and abdomen must be perverted; in consequence of which, dyspnœa or asthma, a livid colour of the lips, cheeks, &c., with some swelling of the hands and feet, frequently occur. The same causes operating upon the contents of the abdomen, produce dyspepsia, costiveness, and occasionally the functions of the liver are impaired and torpid; nor are the kidneys and bladder exempt from morbid affections, a paralysis of the sphincter vesicæ being occasionally produced: but the practitioner need scarcely require the various anomalous symptoms produced by these causes to be pointed out to him; it is only necessary to say, that excurvations of the cervical vertebræ present one or two peculiarities,—the first of which is, that the patient frequently finds himself unable to keep his head erect, and consequently reposes it on a table or any thing before him. There may be in such cases, also, numbness and loss of the voluntary motions of the upper extremities; the pain and difficulty in rotating the head is great, together with the oppression in breathing.

Chapter III. *On the remote and immediate causes of curvatures of the spine, and of predispositions.*—Our author commences this chapter by expressing his surprise at the belief, which has existed since the days of Mr. Pott until recently, that all cases of curvature of the spine originate in caries, and that, wherever there is caries, there must have existed scrofulous action in the bodies of the vertebræ, as the sole cause of disease. Now, observes Mr. Bampfield, curvatures *without* caries are of more frequent occurrence than those *with* caries; and the causes of this disease are so numerous, as to require to be divided into remote and proximate: the most prominent of the former of this class of causes, are falls and contusions on the spine, syphilis, careless and habitual malpositions of the body, strains of the back, rheumatism, rachitis, and mollities ossium. Now, we must confess that we feel inclined even to narrow this list; since what has been called syphilitic caries of the spine we conceive to be more attributable to scrofulous diathesis, in those cases where mercury has been freely exhibited: and, after all, it must be admitted that, although contusions and sprains do occasionally give rise to distortion, it is only where scrofula exists in the constitution, and is ready to be excited whenever a part is weakened by disease, or has been the subject of accident. But it has also lately been taught that curvatures of the spine originate frequently in relaxation of the vertebral ligaments, gradually producing a luxation, or subluxation, of the vertebræ.—We cannot but feel gratified that Dr. E. Harrison's opinions were given to the world through the medium of our pages, since they have brought this long-disputed point to the test of experience.

We should do injustice to Mr. Bampfield, if we attempted to abridge his arguments upon this contested point; we therefore give verbatim the following passages:—

"Ligaments, says Mr. Wilson, with one exception, possess no elasticity; they therefore do not yield, when pulled, but resist until torn through. The ligaments of the spine are all corroborating, and not loose. (*On the Bones*, p. 69, 72.) It is not intended to deny that relaxation of the ligaments of joints sometimes occurs in adults, reduced in strength and flesh by disease, and great relaxation of the ankle, wrist, and knee-joints, occasionally occurs in children, by which their extent of rotation and movement is greatly increased; and, in relaxation of the ligaments of the ankle-joints, the patients are often rendered incapable of walking on the flat sole of the foot, but are under the necessity of walking on one side of it, more particularly on the inside; and Sir A. Cooper has lately recorded two instances of dislocation of the patella from the same cause.* Still, unless future facts and experience shall convince me of the contrary, I shall distinctly deny that either permanent curvature of the spine, or luxation of the vertebræ of the spinal column, is caused by relaxation of the vertebral ligaments. First, because the joints of the spine are secured, not by one, but by many ligaments; and dissection has not yet disclosed to me that relaxed condition of the vertebral ligaments, which can be justly considered the cause of curvatures of the spine, or of the vertebral 'bones not holding together.' In my own dissections it has not been observed; and, in the dissections of Dr. E. Harrison's two cases which have been recorded, (and they are the only two of his,) no such appearance is mentioned.

"Secondly. Let it be admitted, for argument's sake, that relaxation of ligaments does exist, and allows of an extensive play and motion of the vertebræ on each other, still it would not necessarily follow that such a state should be the cause of spinal curvature: for it has been

* Sir A. Cooper, Bart. on Dislocations, p. 11, 12.

stated, and can be clearly demonstrated, that no permanent curvature of any part of the spine can be induced, as long as the bodies of the vertebræ and intervertebral substances preserve their natural dimensions; and this they could and must do, if the disease be confined to relaxation of ligament.

"Thirdly. If relaxation of ligaments were the cause, it would naturally result from the superadded powers of increased and extended motions of the vertebræ, the same as is observed in the ankle-joint, that, if the bones could be displaced and curved with facility, from the ligaments not holding them together, they could also be at once replaced in their natural line, with the same ease, by mechanical force, and the crooked soon made straight. But the fact is, I have never seen a case of permanent curvature of the spine, in which the projecting and arched bones could be reduced, at once, to their proper spinal line, by any force or means whatever; nor a case that gave satisfactory evidence of relaxed ligaments and loosened vertebræ.

"Fourthly. When several bodies of the vertebræ are destroyed by progressive or ulcerative absorption, what powers prevent the spine from separating or dividing at this point?—or, to adopt the phraseology of the abettors of the doctrine we impugn, what holds the remains of the half-destroyed bones together? The answer must be, there is no other competent power than the ligaments, which are somewhat assisted by the muscles; but it is a well-known fact, that the muscles will not hold a joint together after the ligaments are rendered incompetent, or are destroyed. The ligaments are first assisted by the muscles, and subsequently much more so by the anchylosis of the spinous processes, and sometimes of the other articulatory processes.

"Fifthly. If the spinal ligaments were relaxed and elongated, the practice of very forcible extension of the spine by machinery would be very injudicious and pernicious, as it would tend to loosen and elongate them further; yet the physician alluded to always employs this remedy.

"Lastly. If the doctrine were true, Avicenna and Dr. Glisson would have a long priority of claim to it.

"Having denied the premises, it follows that I cannot admit the consequences, and reject the easy transition from relaxed ligament as a cause, to luxation and subluxation of the vertebræ as effects:—First. Because neither dissection discloses, not even in Dr. E. Harrison's own cases, nor do the preparations in the anatomical museums display, either of those states after death. 2dly. Because we have not seen either state of luxation in any case of distorted spine in the living. 3dly. Because we are sceptical enough to believe that complete luxation of the spine must necessarily be followed by death, notwithstanding Dr. E. H. states that, on examining his patient, F. G. Pratt, of Camden Town, ten months after the accident occurred, he 'found the first lumbar vertebra wholly dislocated, and driven into the left loin. The last dorsal and second lumbar were also displaced,' &c.* and subluxation

* "See *Medical and Physical Journal*, for December 1820, p. 449.—This boy is since dead; and, on examining him, Mr. Shaw discovered it to be a case of must be followed by pressure on the spinal marrow, producing paralysis of the parts inferior, convulsions, and even death. Indeed, if relaxed ligaments admitted of the extended play of the vertebræ on each other, we might induce paraplegia, and remove it at our pleasure, by causing subluxation and pressure on the spinal marrow. 4thly. The best and greatest authorities, Sir A. Cooper, Pott, and Boyer, declare there is no dislocation. 5thly. In those cases where several of the bodies of the vertebræ are destroyed by progressive or ulcerative absorption, and in which the dislocation of the processes would be the easiest, luxation has not been induced, or, at least, not recorded: but Nature, always wise, always preservative in her efforts, prevents such a result, by forming an anchylosis of the spinous processes, that must be broken before any dislocation can ensue." (P. 61—65.)

The only remaining portion of this section which we need notice, is the opinion maintained by our author, that debility of the muscles of the back, whether congenital or induced by disease, is a frequent predisposing cause of permanent curvature of the spine; for though, at an early period of this form of the disease, no structural change takes place in the vertebræ, yet, if the patient be neglected, this will at length ensue.

The proximate causes of curvatures are next considered, in the second section, at considerable length; of which the reduction of the bodies of the vertebræ and intervertebral substances, from a state of equal thickness to a cuneiform shape, is the most frequent, and is the product either of ulcerative absorption or progressive absorption, without caries or the formation of pus, —of an irregular growth of bone,—or, finally, of either of the above affections of the intervertebral cartilages. Our author illustrates his meaning in the following manner:—

"As long as the constituent parts of the vertebral column accurately maintain their relative proportions, agreeably to their natural formation, no permanent curvature or angular projection can take place. If, in this state, a wedge were applied between any two vertebræ on any side, the spinal column would be forced out of its spinal line, and incline to the opposite direction.

"Thus, if it were applied posteriorly, it would cause it to deviate from its spinal line, and bend forwards, and *vice versa;* and if the wedge were driven in between two vertebræ on the right side, it would occasion the column to incline over to the left side, and *vice versa;* and, were it not for the retaining powers of the muscles, ligaments, &c. the spinal column would be dislocated or overthrown altogether. In this point of view, as well as in those we have already taken, of the effects

---

'fracture in a horizontal line in the middle of the first lumbar vertebra, the lower half continuing attached to the other lumbar vertebræ, the other to the dorsal. The two portions are united together obliquely by a ligamentous matter, so that, at this part, there is an appearance of dislocation; but there is not the slightest displacement of any of the other vertebræ. The spinal marrow is completely destroyed at the fractured part.'" (P. 77, of Mr. S.'s work.)

of ulcerative or progressive absorption of the horizontal surfaces of the vertebræ and intervertebral cartilages, permanent curvatures, or angular projections of the spine, are merely mechanical consequences; and if we can demonstrate that a power equal to, and acting on the principle of a wedge, be applied in such cases as a cause, I shall prove the position just laid down. Here I must recur to the old exploded doctrine of unequal growth, or malformation of bone, which has been assumed, by Glisson and others, as an efficient cause of distortion; a doctrine exploded, too, against the evidence of the most perfect of our senses: for, in the anatomical museums, specimens of deformity arising from the unequal and irregular growth of all the bones, and of course of the vertebræ, may be seen; in some of which, the malformation or inequality is produced by diseased actions, but, in others, simply by an exuberance of the 'formative principle,' or by an error of nature, in exciting a pruriency, or occasioning a deficiency of growth. It can only be intended, however, to offer illustrations of the effects of this error of nature in the vertebral column; and this I am enabled to do in a manner that appears satisfactory, by resorting to the appearances which dissection discloses in H. Pittam's and other cases, as A. Selby's, and examining the preparations of the various distortions of the spine, that are placed in the Hunterian, Brooks', and other splendid collections of morbid anatomy. In viewing the preparations, and in the dissection of spinal deformities from irregular growth of bone, the disproportions of the different bodies and intervertebral cartilages,—that is, between their outer and inner lines of curvature, are most striking; the thickness of the vertebræ and intervertebral substances being sometimes, on the outer line of curvature, double, or even treble, of that of the inner line. It therefore remains to be explained by what process this immediate cause produces its consequences.

"When an increased growth of bone takes place on one side of the spinal column, which is a flexible one, it must necessarily elevate the parts above it on the same side, and make them recline over to the opposite side, which is thinner, and that must now sustain the greater share of weight; one consequence of which must be, that a greater degree of pressure will be made by all superincumbent weight on the thinner side, to which it is reclined, whilst it will be diminished on the side which the increased growth has elevated: and, indeed, whilst the superincumbent weight will press the surface of the joints more closely together on one side, it will tend to open the joints, or separate their surfaces, on the other, and put the ligaments on the stretch, and (as it were) create a space for interstitial deposition.

"Now pressure is the strongest power we possess of producing local absorption; and, as increased growth of bone is a cause of a permanent nature, the pressure too becomes permanent, (except in particular positions of the body,) and occasions absorption of that part of the bone, and of the intervertebral cartilage, unduly pressed upon. Thus, increased growth on one side, and progressive absorption occasioned by pressure on the opposite side, both conspire to the same end, and both operate in destroying the natural proportions of the vertebræ, and of deflecting the erect spine from its spinal line to the form of a curve; and, in the degree that increased growth takes place on one side, and increased pressure and absorption on the other, the greater or less must be the curvature. Besides, as these causes extend their effects, the number of the vertebræ involved in the curve will be increased, until many are bent into an arc, some of which have had their dimensions increased on one side, and all of them have been diminished on the other; which is a very important fact to be borne in mind in the treatment. During this deforming process, we may reasonably believe the muscles and ligaments offer some resistance to its progress, but effects show their resistance is overcome." (P. 74—77.)

In the same way the formation of the angular projection, and the excurvation of the spine, may be explained. Our author also remarks, that he has met with cases of excurvation which could not be fairly attributed to any other cause than rāchitis; in which case, it would appear that the superincumbent weight and pressure are not always necessary to the production of curvature, and that *mere position* will not counteract it.

Two circumstances chiefly conduce to the formation of excurvations by the irregular growth of bone; the increasing enlargement of the posterior portions of some vertebræ, and the absorption of the anterior portions by mechanical pressure; besides which, as the curvature increases, the evil will be augmented by the weight of the parts above the curvature, not being borne equally by the whole spinal column, but by their being thrown on the posterior part of the inferior vertebra, which still preserves the spinal line. It must be recollected that, though in some constitutions progressive absorption may go on without inflammation or ulceration, yet that pressure may produce them both; though the ulcerative absorption is much less frequently induced by it.

Chap. IV.—The fourth chapter commences with a section on the *prognosis* of these affections: this may be favourable as far as relates to the preservation of life, where there is no scrofulous diathesis, and when the treatment is begun at an early period. The question of the restoration of the spine to its pristine and natural form, is totally distinct. In curvatures caused by caries, the prognosis is unfavourable; but more especially so if combined with abscess, whatever situation it may occupy. In curvatures from progressive absorption, unless the health be much deranged, a favourable termination may be anticipated. If these diseases are attended with frequent head-aches, they are dangerous; with hectic fever, they are fatal. Palsy of the lower limbs indicates danger, but such cases frequently recover.

Section 2d, on the *diagnosis*.—As the permanent deviation of the spine from its natural line of conformation is a sufficient mark of the existence of the disease, Mr. Bampfield devotes this section to a consideration of those symptoms which distinguish a curvature with caries from one without, as well as to those symptoms which ought to awaken suspicion, and lead to an examination of the spinal column. Of these latter circumstances, a sense of constriction across the epigastrium, disordered respiration, a sense of weakness in the back, and a habit of stooping or leaning to one side, are the most common. In curvatures with caries, there is more pain, more fever, and general constitutional disturbance: this form also produces pain upon exercise. There will frequently be found conjoined with it affections of the joints and swellings in the glands, great mental irritability; and, finally, as pus becomes formed, cough and hectic fever supervene. The inflammation, ulceration, or gangrene of the intervertebral substance, appears to our author to be attended with more local pain and more general suffering, than caries of the bone itself. These diagnostic signs are indeed but unsatisfactory, inasmuch as they do not announce a *coming* disease, but one already formed; and therefore we are prepared for our author's remark, that there are no *certain* diagnostics of an incipient curvature.

"When, however, a patient complains of any pain in the course of the spine, attended with a sense of constriction across the epigastre, and an occasional feeling of numbness or twitching of the upper or lower extremities, and is relieved by bending backwards, or to one side, or by lying down,—suspicion should be roused, and the back should be inspected. If any disease should exist, the dorsal muscles are generally wasted; if any degree of curvature exists, the projecting spinous processes will demonstrate it; if of inflammation, pressure and the application of the hot sponge will excite pain. In curvatures without inflammation or caries, the hot sponge as often affords ease or comfort as it produces any other sensation.

"In an incipient excurvation, the undue prominence of any of the vertebræ, or rather of their spinous processes, will be most displayed by bending the body forwards, in which position any unnatural projection becomes evident." (P. 89, 90.)

Chap. V.—We have now arrived at the *treatment of excurvation*, premised by some remarks on the means of prevention, and the indications of cure; and these occupy the first section of the fifth chapter. When the spine has begun to acquire a bent position, either in consequence of rheumatism, contusion, strains, or simple inflammation, the recumbent position is the most obvious remedy. Malposition of the back from bad habits, may also be obviated by the same means, or by proper instruments to support the spine.

Of the indications of cure, some are general, others are applicable to one variety only. The first indication is to relieve the spine from *all* superincumbent weight; the reason of which is so obvious, as not to require a comment. For the purpose of taking off all pressure from the vertebræ, two methods have been proposed,—the recumbent posture on a horizontal or an inclined plane; and our author demonstrates the superiority of the horizontal plane very satisfactorily, since it is impossible to relieve the spine from *all* weight on the inclined plane, the quantum of weight which it will have to sustain being exactly in proportion to the angle of inclination of the plane. The second indication is to abstain from all motion whilst lying in the recumbent position; and of that position our author recommends three varieties, as applicable to the treatment of these diseases,—that is, lying on the back, the facial-horizontal position (which is the reverse of this), or lying on either side. In each and all these cases, the general health will demand the same degree of attention to the articles of diet, air, hours of rest, &c. &c.

Section 2d, on the *treatment of excurvation of the spine.*—We now come to the discussion of the author's peculiar views in the management of these cases. Whilst he advocates a recumbent posture on a horizontal plane, he argues against the position heretofore employed, and recommends in its stead the adoption of the facial position of the body; and he gives the following reasons for this preference:—In the first place, in this position the spine naturally tends to fall inwards, and thus to diminish the excurvation; 2d, all superincumbent weight is taken off; 3d, in this posture, pressure, friction, or extension may be employed, and issues and setons, &c. may be easily managed; 4th, the projecting sternum is pressed towards the vertebræ by the effects of superincumbent weight; the position is also more agreeable, as he is to be placed on a soft feather-bed, and enjoys some little freedom of motion; 5th, he can, in this position, also eat without inconvenience: some minor advantages are also enumerated.

In conjunction with this position, the benefits and effects of mechanical extension and pressure are next considered; but we must pass by our author's reasonings on this point, and proceed to state the method of employing them:—

"In the incipient stage, extension of the vertebral column may be employed as the patient lies on his bed, either by machinery made for the purpose, on the principle of a windlass, or by one assistant pulling at the lower extremities, whilst another pulls by grasping the wrists, or under the axillæ; or, where assistants are wanting, the patient may pull with his hands grasping the bed-posts, whilst an assistant pulls the legs.

"During this extension, pressure should be applied, with the hand covered with a glove, (or a compress may be laid between the hand and skin,) on each projecting spinous process individually, or on the whole at once; or it may be applied to the transverse processes and ribs on each side, when the skin is tender over the spinous processes, or it may be employed alternately in both situations. In the advanced stages of excurvation, the patient has been generally placed on pillows laid on a table, between two of which a space has been left corresponding with a line drawn from the middle of the curvature, and in this eligible position extension and pressure have been used. These two mechanical powers have been generally employed as long and as firmly as the patient could endure it without fatigue or complaining of pain, and the power of the pressure, in particular, has been graduated by the feelings of the patient.

"Extension and pressure may be repeated frequently during the day, without disturbing the apparatus to be applied, and to be presently described.

"After friction, extension, and pressure have been employed, the apparatus of a compress, a quilted pad, made of what ladies call wadding, covered with silk or cotton, and a shield placed over the curvature, and secured by a long broad bandage, rolled round the thorax, should be applied, as the patient lies on the pillows, which is the most eligible method; or he must be raised on his knees and elbows, so as to form a space under him that will allow free liberty to pass the bandage with the hand. The bandage should be rolled as much as possible during expiration, when the ribs are not distended or raised; because the bandage would be then firmer, and because the power of inspiration, by raising the ribs, would tighten the bandage on the projecting sternum and vertebræ, and be almost a natural means of mechanical pressure. Should inflammation of the vertebræ be present, attended with pain, or if the mechanical operations excite pain in any degree, much complained of, they must be suspended or desisted from, until the inflammation has subsided, and the operations cease to occasion pain." (P. 109—112.)

The utility of setons or issues is next discussed. Our author appears to believe that the number of cases in which they are beneficial, is not so great as has been usually imagined, and that they are more frequently indicated in the excurvations of adults than of children.

On the medical treatment we do not conceive it necessary to dwell, since, though what our author advances on that subject is highly judicious, it is no more than what is to be met with in other works of this description.

With respect to the time which it is necessary to employ in the use of the above means, we can only observe generally, that much depends upon the extent of surface diseased. The work contains some very sensible directions on this point; but we must proceed to notice another circumstance of paramount importance,—the time when, and the conditions under which, active exercises of certain classes of muscles may be employed in spinal curvatures; for it is obvious that, if it be made use of whilst active inflammation, ulceration, or caries, be going on, that nothing but evil can ensue: it therefore must be restricted to cases of debility of the muscles, to those of rickets, of malposition, of disproportionate growth of bone, or, finally, when the patient is recovering from a state of curvature, by regeneration of the bone, after progressive absorption of it and the cartilage. We regret that our limits will not permit us to do more than merely recite some of the modes of exercise proposed by different surgeons, beginning with Mr. WILSON's plan of carrying a weight upon the head, which seems chiefly adapted to incipient or slight cases of curvature, but, as it implies an upright posture, cannot be generally adopted; and terminating with Mr. JUKES' chest-dilator, of which a plate is given. Of course, Mr. WARD's and Mr. SHAW's methods of giving exercise to the muscles of the chest and spine are noticed, they are likewise explained by diagrams. It is to be observed, that all these methods must be followed by intervals of complete rest.

Having discussed this subject, Mr. Bampfield proceeds to consider the much-contested subject of collars, and other mechanical means for the support of the spine, which have been so warmly condemned by some, and approved by others. Our author appears to be disposed not altogether to reject their employment, though, in stating his opinion, he seems to apprehend that he may incur the imputation of being called a "trimmer;" but he gives us in the following passage a summary of his opinions respecting the use of machinery:—

"In adverting again to the differences of opinion which have existed respecting the utility and propriety of employing mechanical apparatus for the cure and prevention of distortion, I would first observe, that they should not be trusted to alone in any case, as is too much the practice, where the patients apply to machinists who are not surgeons, or where the surgeons consulted have not turned their attention to the subject, and refer the patients to machinists: but the mechanical apparatus, of some form or other, can be employed with advantage in some stages of the complaint, provided their use only constitutes a part of the treatment, and is combined with such other means as have been described and recommended in the different stages of the disease.

"I so far coincide with those who oppose the use of instruments, in believing them to be unnecessary and useless in those stages of spinal disease in which I have stated the use of the horizontal posture to be indispensably requisite: indeed, when circumstances or symptoms render that posture advisable, the use of instruments is improper; and, if employed as a substitute for it, the practice would be highly censurable,—for no instrument, or combination of instruments, could so effectually relieve the spine from superincumbent weight and pressure as proper horizontal position: as well might the surgeon, in a case of scrofulous inflammation of the knee-joint, put on an instrument to restrain its motion, and desire the patient to walk about on it! The use of instruments—for instance, of the collar,—will not relieve the disordered respiration, whether dyspnœa or asthma; their use will not prevent the accession of fits or chorea; neither will they conduce to the cure of paraplegia, nor remove the sense of corded tightness across the epigastre, so effectually as proper recumbent posture; and, indeed, they frequently fail in those objects entirely, and the patient gets worse under the use of the collar.

"Instruments for stretching and supporting the vertebral column, should not be employed during scrofulous or simple inflammation, or caries, or vertebral destruction by absorption; neither should they be resorted to during the regeneration of bone. During inflammation, the stretching of the ligaments would irritate and probably increase it, and the support from the collar would not be effectual in taking off all superincumbent weight and pressure. During or after extensive destruction from ulcerative or progressive absorption, the remains of the vertebræ might be fractured by forcible stretching even by the windlass, and much more likely by the mode of stretching the spine by suspension by the head." (P. 149—151.)

After the proper spinal line has been restored by the means recommended above, the collar, or spine supporter, has also been found useful by the author.

Here we must pause,—with a promise, however, to continue our review of Mr. Bampfield's work in our next Number. It is a subject which requires to be discussed at some length, and cannot, with justice to the author, be compressed in the compass of a single article.

---

### DIVISION II.

### FOREIGN.

ART. II.—*Observations Pathologiques propres à eclairer plusieurs Points de Physiologie.* Par F. LALLEMAND, Professeur de Clinique Chirurgicale a la Faculté de Montpellier. Deuxieme Edition. —A Paris, 1825. 8vo. pp. 143.

M. LALLEMAND is already favourably known to the profession, by the sedulous attention he has paid to physiology and pathology. The present work has been published for some time, but, from its having been out of print, we have not till now, on the appearance of a second edition, been able to lay an analysis of its contents before our readers.

The opportunities enjoyed by the author of making pathological observations in an extensive hospital, in which he resided as medical officer, led to the publication under review; the particular object of which is to clear up some of the doubts which still perplex our investigations upon many physiological questions, and at the same time to offer some suggestions in opposition to opinions which are generally admitted as incontrovertible, and which are chiefly founded upon experiments instituted upon animals. For ourselves, we always view with an eye of considerable distrust any physiological doctrines that rest entirely on such a foundation. We do not deny that many valuable facts have thus been added to our store of knowledge; but we are equally convinced that many erroneous opinions,—and we fear also many practical errors,—have been promulgated from the adoption of partial and hasty decisions. The great difficulty that must always exist of drawing a parallel between the healthy functions of a part, and those actions which meet the eye of the experimenter in animals writhing with torture, or stupified by artificial means, must always render such investigations unsatisfactory, if not altogether nugatory. We have on other occasions stated, and now repeat, that it is far from our wish to offer any opposition to well-imagined and skilfully-executed experiments upon the lower animals, in order to ascertain points which cannot be determined by any less objectionable means. It is doubtless a source from which much valuable information has been, and may be, derived, provided the imagination of the operator is not led astray by opinions which have been previously formed in the closet, and which *it is hoped* the experiments will verify. Even with regard to comparative anatomy, it is true that it enlarges our ideas, and conducts us to general results of great value; but, before we apply these analogies to the economy of man, they ought to be confirmed by facts derived from our observations of man. Now one of the objects of M. Lallemand corresponds with this view, as he endeavours to prove that we derive, from attention to pathology, as much physiological knowledge as from zoology, or vivisections; and that much more reliance may be placed on the information thus obtained.

The facts related in the chapters we are now about to glean for our readers, have been witnessed by the medical officers of the Hôtel Dieu and the numerous pupils attached to that establishment: they commence with a case of extra-uterine conception, which is, in some respects, curious.

Maria T—, thirty-five years of age, by trade a sempstress, of a feeble and irritable constitution, and so jealous a disposition, that she was constantly in a state of anxiety about her husband; and moreover of a very amorous complexion, *(et lassata viris, nedum satiata.)* She menstruated for the last time at the end of September 1815. At the beginning of October, "les deux epoux furent un jour surpris immediatement apres le coït, par l'entrée d'une personne du voisinage, qui ouvrit brusquement la porte." So unexpected and unwelcome an intruder produced, as might be expected, considerable embarrassment on the part of the lady; she became speechless and extremely agitated. On the following day she suffered from pains resembling colic, and also from a fixed pain in the left iliac region; these pains increased from day to day, and, after much suffering, she discharged from the vagina a clot of blood, mixed with a reddish serum. During three months she made use of a variety of remedies, without effect; and, at the commencement of January, the pains, which had at first been seated only in the left iliac region, occupied every part of the abdomen: they were still, however, more severe in the left side than any where else. The belly gradually enlarged; the fæces being at the same time passed with pain and difficulty. In this state she was admitted into La Pitié, on the 20th of January, 1816. It was imagined by M. GEOFFROY that she was labouring under chronic peritonitis, and that some organic disease of the abdominal viscera existed. The treatment adopted was ineffectual; the pains gradually increased; she became much emaciated and altered in her appearance. During the last few days of her existence, she screamed and groaned incessantly, and died the 30th of March, 1816.

*Inspection of the body.*—Upon opening the abdomen, the capillary system of the intestines, and of the peritoneum covering the parietes of the pelvis, was found in such a state as to warrant the inference that very violent inflammation of these parts had existed. The colon on the left side, and its fatty appendages, were of a violet colour; the lower part of the great omentum presented the same appearance. The uterus formed a tumor projecting above the pelvis, and was about double its usual size. An incision having been made upon the anterior part, in its cavity was found a soft, pulpy, retiform, reddish substance, similar to the erectile tissue of the corpus cavernosum: this substance was united to the uterus, the cavity of which it entirely covered by a sort of "*tomentum*," which was easily torn. It formed a cyst, the parietes of which were about one line in thickness; its cavity (from four to six lines in diameter) was smooth, without any opening, either opposite to the cervix uteri or near the fallopian tubes. To the left ovarium, and to the broad ligament of the same side, adhered a spongy mass, which extended from thence to the sigmoid flexure of the colon, and to the posterior part of the uterus, which was covered anteriorly by the inferior part of the great omentum. This spongy mass had, in many parts, a resemblance to the false membrane produced by inflammation of serous surfaces. Other portions of it, when carefully examined, so closely resembled the placental structure, that this was the first comparison which occurred to the minds of the persons present. Having laid aside the omentum and the colon, to examine the interior of the pelvis, a sac filled with water, in the midst of which floated the feet of a fœtus, apparently about the sixth month, was discovered; the body and head being concealed at the bottom of the pelvis. The quantity of serous fluid which escaped was about twelve ounces. The flesh was firm, and the skin was covered with the usual unctuous matter; every part was well formed, except the cranium, which was flattened and "contourné," in consequence of the obstacles which had existed to its more perfect development. The umbilical cord, which was eleven inches long, was inserted upon the edge of the placenta by several large vessels The chorion and amnion were distinct, and easily separated from each other, lining almost the whole cavity of the pelvis: the chorion adhered to the surrounding parts by means of a flocculent tissue, which was red and vascular. In carefully detaching this false membrane, an immense number of very minute vessels were discovered shooting from the peritoneum, to lose themselves upon its external surface. Other similar vessels united its internal surface to the chorion. Towards the circumference of the placenta, which was about five inches in diameter, and seven or eight ounces in weight, this membrane became firmer, thicker, and of a darker colour, presenting exactly the appearance of those clots of blood which are found in old aneurisms,—homogeneous and easily torn. In some parts it was about a line in thickness.

The peculiar circumstances of this case are curious: it is seldom we have an opportunity of catching nature so nearly in the fact; and we can here follow her operations, and trace the successive phenomena, step by step. The observations of M. Lallemand upon the case, are deserving of attention. It is very true, however, that all such deviations from the usual course of nature afford more opportunity for ingenious speculation than solid demonstration: we may conjecture, but we cannot know.

"It was at the latter end of October, 1815," says our author, "that this couple were surprised immediately after coition. On the same night commenced the development of those symptoms which they attributed to alarm. The death of the woman took place on the 30th of March, being an interval of six months. It has been observed, that the dimensions of the fœtus announced that term of existence; and, from the dates connected with the other circumstances of which we have spoken, fecundation of the woman can only be attributed *au moment precis du coït en question.* It appears evident that the alarm which was experienced produced a general relaxation of all the tissues of the economy, and destroyed the erethismal state of the fallopian tubes, (for it is particularly upon the erectile tissue that its effects are excited;) and that the ovum, in detaching itself from the ovary, not meeting the canal which ought to have conducted it to the uterus, fell into the cavity of the peritoneum. It is, besides, easy to demonstrate that the ovum must have quickly detached itself; for, after having experienced much uneasiness during the night, the patient complained next day of a fixed pain in the left side of the abdomen. Now, we know from positive experience (Bichat) that the serous tissues are not sensible until after having been inflamed for a longer or shorter period."

It is an admitted fact, that the uterus is brought into a state of activity whenever impregnation takes place: it increases in volume, and its cavity is dilated, wherever the development of the fœtus is effected. These circumstances are witnessed in every case of extra-uterine pregnancy.

"The membrane we found," continues M. Lallemand, "between the peritoneum and the chorion had not descended with the ovum, since the membrana decidua was found in the uterus; it had, therefore, been accidentally developed upon the surface of the peritoneum, in the same manner as false membranes,—that is to say, by the effects of inflammation. The disease presented all the appearances of peritonitis; the ovum had certainly been the cause of this inflammation: it had acted upon the peritoneum as a foreign body,—with this difference, that, being endowed with life, it was enabled to form adhesions with that false membrane. It was shaggy, more vascular, and thicker opposite the placenta, than in any other part. The membrana decidua offers precisely the same disposition towards the sixth month of pregnancy. Again, we cannot refrain from assimilating this membrane to those which are formed upon serous surfaces in the process of inflammation. This adventitious membrane of the ovum is, therefore, a natural transition, which conducts us from ordinary false membranes to the deciduous membrane, and explains its formation and uses. It is not therefore, as has been imagined to be, the product of an exfoliation of the mucous membrane of the uterus, nor the degeneration of the sperm in its cavity.* We must admit, with William Hunter, that the decidua is formed by means analogous to those which produce other false membranes. We know that the uterus, after conception, is in a state of turgescence nearly approaching to inflammation. So striking, indeed, is the resemblance, that Mr. Harvey, after experiments upon dogs, compares the uterus at that time to the lip of a child stung by a bee. This appearance cannot be attributed to the presence of the fœtus in the uterine cavity, since we find the deciduous membrane in every case of extra-uterine conception. It is very probable that it is the contact of the seminal liquor within the surface of the uterus, which determines so important a change in its vital properties."

We must refer to the work itself for some ingenious suggestions upon the frequently-contested point of the kind of communication which exists between the mother and fœtus. The opinion of HALLER has long been abandoned: in this instance it is probable that this great physiologist drew conclusions from experiments not instituted by himself. Whether the opinion

---

* M. ROUX, *Anat. descriptif de* BICHAT, t. v. p. 368.

offered by M. Lallemand will ever attain the rank of a correct theory, is yet to be determined: we think it will not. That nature should institute a state of disease for the purpose of completing the grand operations which are necessary to the perfection of the fœtus, does not appear probable; although we must confess there is much plausibility in the observations of M. Lallemand upon this subject.

The relation of this interesting case is followed by a brief article, under the title of "Communication between two Placentæ united into one mass, in some cases of Twins." Although it is stated in the works of LEVRET, BAUDELOCQUE, GARDIEN, CAPURON, MAIGRIER, &c. that in cases of twins, when the placentæ are united into one mass, there is no continuity of blood-vessels,—and although the exceptions to this statement are few,—it is, in this country at least, considered incumbent upon the accoucheur to act as if a vascular communication did exist. Two ligatures are, or ought to be, invariably placed upon the umbilical cord before it is divided. This precaution can never be attended with any ill effect; and, if it should happen that, in a case of twins, there does exist a vascular communication between the placentæ, (which may be the case, although they be apparently united into one mass,) the life of the second child might be placed in danger without it. It has been noticed by SMELLIE, that in some cases twins have but one placenta: he injected the artery of one umbilical cord, and the fluid passed into the vessels of the other. Professor DESOR has recorded a similar fact.* With respect to the article before us, we find it to be little more than an iteration of the opinions stated in the Dictionnaire des Sciences Medicales, art. *Placente*, tome xlii.

The next paper in M. Lallemand's collection is an account of the Distribution of the Nerves in an Acephalous Child: it is not devoid of interest, but our limits compel us to omit it, and pass on to that which follows, entitled "Observations on the Functions of the Digestive Organs,—on Vomiting." No explanation has yet been offered of the action of vomiting, which has not been deemed erroneous in some of its parts, by those who have paid much attention to the subject. It is true we know that several organs are actively engaged in completing the phenomena of vomiting; but the question is yet *sub judice*, what is the precise part which each particular organ performs? We are not called upon to enter into an elaborate statement of the opinions which have been advanced, contradicted, and reasserted, upon this important subject. We have only to present to our readers the substance of M. Lallemand's remarks, which

---

* *Journal generale de Medecine, &c.* July 1816.

are valuable and ingenious. In pursuance of the custom which our author has prescribed for himself, and which many others would do well to imitate, he commences by the relation of pathological facts, and then—and then only ventures upon speculation.

"A woman, of strong constitution, had suffered suppression of the menses, in consequence of fright and imprudent conduct. For six months the menstrual discharge had not appeared: during this time, at each monthly period, she had had an attack of hematemesis, which lasted three or four days. When the process of digestion commenced, she experienced a shivering or coldness of the extremities, considerable heat in the epigastrium, and, at the end of half an hour, the congestion produced in the stomach brought on a flow of blood; of which she was conscious, from the cessation of the unpleasant sensation of heat, and also from a sense of heaviness and uneasiness, which was quickly followed by nausea and vomiting. It is extraordinary that she never vomited any thing but clots of blood, sometimes of considerable size, which were always perfectly free from any mixture of food or drink, which had been previously taken. At the termination of about half an hour, the vomiting ceased, the patient became calm, and digestion continued as if she had been in the enjoyment of perfect health. M. Recamier, in his clinical observations upon this woman's case, related several facts, which prove that the stomach exerts an elective action upon particular substances,—that it possesses the power, during the efforts of vomiting, of retaining some and discharging others."

We may observe that the pylorus is endowed, to a certain extent at least, with an analogous power. The structure of the pyloric extremity of the stomach is so contrived, that some part of the mass contained in the stomach is allowed to pass, whilst others are retained until digestion is more fully completed in them. In the glandular system, we also see many examples of similar elective power. The excretory duct of a gland will not admit the fluid that is secreted, until it has undergone certain necessary changes in the parenchymatous structure of the gland. In most cases of vomiting, however, the stomach does not exert the power of expelling only a part of its contents. We have in our recollection several cases of hematemesis occurring in females, under circumstances very similar to those detailed by M. Lallemand, in which the blood thrown up was always mingled with such articles of food or drink as had been previously taken.*

We know not whether the question has ever been particularly discussed, but we believe it to be currently admitted that the stomach does possess the power of rejecting what is offensive

* Does hematemesis frequently occur in the male, independent of external violence? Is it not always connected, in the female, with some disturbance of the menstrual discharge?—Rev.

to it. For instance, we are acquainted with an individual who will not bear particular articles of diet, which happen unfortunately to be agreeable to his palate: when he indulges in such articles, he pays the forfeit of his folly by an attack of vomiting some hours after; and it frequently happens that the obnoxious matter alone is rejected, although the stomach contains several other articles of food.

M. Lallemand next relates a case, in which, during efforts to vomit, a rupture of the stomach took place. He doubts the accuracy of the experiments which were instituted not long ago by M. MAGENDIE and others, for the purpose of proving that the stomach is not actively engaged during vomiting.

The last chapter, or rather division of the work, contains some observations upon the action of the stomach upon different kinds of food. The importance of this investigation requires no proof: it has occupied the attention of many able men, among whom we hesitate not to class the author before us. M. Lallemand has derived his information upon the subject, from watching several patients who had artificial anus. The great success obtained by M. Dupuytren in the management of such cases, drew many patients to the Hôtel Dieu; and the conclusions drawn from his attention to these cases leads him to infer—

1st. That, if it be true that articles of food the most animalised are those which are most nutritious, and *vice versa*, it does not follow that they are the most quickly digested.

2dly. That, on the contrary, the digestive process is long and difficult, in proportion to the quantity of nutritious matter contained in a given quantity of food, and *vice versa*.

3dly. That articles of food are not passed from the stomach in the order in which they have been introduced; that it is not those which are first altered by digestion which first pass off; but that it is those which, containing the least alimentary materials, offer the least opposition to the digestive powers.

Our opinion of this little work of M. Lallemand, may be inferred from the space we have allotted to its consideration: he is not one of that numerous class of practitioners who observe the progress of disease with indifference; but he appears desirous to glean practical information from the cases entrusted to his case, and possesses the talent of imparting to his brethren the result of his experience in a useful and unassuming form.

Art. III.—*Manuel d'Anatomie Générale, Descriptive et Pathologique;* par J. F. Meckel, Professeur d'Anatomie à l'Université de Halle. *Traduit de l'Allemand, et augmenté des Faits nouveaux dont la Science s'est enrichie jusqu'à ce jour;* par A. J. L. Jourdan, Membre des Académies Royales de Médecine de Paris, &c. et G. Breschet, Professeur agrégè en Exercice, Chef des travaux Anatomiques de la Faculté de Médecine de Paris, &c. Tome troisième.—Paris: Baillière, 1825.

*Manual of General, Descriptive, and Pathological Anatomy;* by J. F. Meckel, Professor of Anatomy in the University of Halle. *Translated from the German, and augmented with such new Facts as are furnished by the Progress of Science to the present time;* by A. J. L. Jourdan, &c. and G. Breschet, &c. 3 Vols.

The high character borne by the author of the above work, as an anatomist and man of science, renders it incumbent upon us to take early notice of his labours; and the rather, as these volumes are so bulky, and contain so much important matter, that we anticipate a succession of analyses, to be given in the manner we shall immediately explain. It is obvious that a work of general and descriptive anatomy cannot, and indeed need not, be scrutinised chapter by chapter, or page by page. It is sufficient to say, that the plan of Bichat is that which our author has followed.

The volume commences with a pretty long chapter upon the general Laws of the formation of the human body, and a description of the Cellular Tissue. We shall therefore, we conceive, perform an acceptable service to our readers by passing this over, and also the first article of the second chapter, which is a description of the Vascular System in a state of *health*, and, by giving them a full account of what the author has to say upon the diseased condition of that system, (pursuing pretty nearly this plan throughout the remainder of the work,) we shall avoid repeating what is universally known in anatomy, and merely present the author's *pathological doctrines* to the consideration of our professional brethren.

*On the diseased Condition of the Vascular System.*

The first of these conditions is *inflammation*, the principal means by which all formations, either regular or irregular, are produced. Inflammation has its seat in the most minute ramifications of the sanguineous system,—that is, in the capillaries, where the blood is accumulated in greater quantity than usual, and is circulated either more or less rapidly than in a state of health. Dilatation of the vessels is the result of this accumulation, and there is also an increased redness in the part affected. When this condition has lasted some time, other phenomena ensue, which are called the terminations of inflammation. But it must be recollected that, excepting in the first instance (that of resolution), inflammation continues, though in a less degree, as long as these consecutive stages last. The most simple of the new formations is exudation, which arises from the extravasation of the colourless parts of the blood, either into the substance or on the surface of the inflamed part, and either producing dropsy, induration, or adhesion: these two latter conditions may exist together. Blood-vessels are commonly formed in the substance effused and coagulated. These are not mere prolongations of those which existed before; they are not so regular; and the serous and mucous tissues are chiefly disposed to these adhesions which impede, in a greater or less degree, the movement of parts. When this action takes place in parts that have suffered a solution of continuity, it is called union by the first intention.

The second species of new formation is suppuration: here the texture undergoes a more essential change, since the inflamed part is converted into a secretory organ of a new kind, analogous to the mucous membranes; but the formation of this secretory organ is not an indispensable circumstance in the production of pus, since it is not to be observed on mucous surfaces. In general, in order that pus may form, it is necessary that inflammation shall have acquired a certain degree of intensity, and lasted some time.

The next termination is sphacelus, which is preceded by gangrene; and this may be either dry or humid. When this condition only produces the death of the part attacked by inflammation, the gangrene stops, and, at the limit between the dead and living part, a red line is formed, the *living* border of which is of a bright red: this line is formed by absorption, which has become more active in order to separate the dead from the living part. The means of separation are much more complicated in the cases of inflammation and mortification, than in those of resolution or adhesion.

Although new vessels are developed in the newly-formed parts, of which some are produced independently of those which existed before, whilst others are only simple prolongations, yet the original vessels have not the power of regenerating themselves entirely. When one of them is destroyed by a ligature, or in any other manner, a new one is never formed in its place. Thus, when a vessel which has been wounded heals by cicatrization, without the obliteration of its canal, that vessel always differs in texture and hardness from those which have not suffered the same accident.

The four portions of which the vascular system is formed,—that is, the heart, the arteries, the veins, and lymphatics, present a number of anomalies, both externally and internally. Among these anomalies, some are common to them all, whilst others belong especially to one of them; and, even of those that are met with indifferently in all, some are more or less modified by the difference which is to be found in the natural condition of each. The deviations from health in the vascular system, may be arranged into those which affect their exterior form, and those affecting their interior formation or texture. The first of these have relation to the situation, volume, configuration, or continuity of the system: they may be congenital, or the accidental product of other causes. The following considerations will be sufficient to give a general idea of the subject.

By anomalies of situation, with respect to vessels, their unusual origin or course is understood; with respect to the heart, its unusual position, &c.

Anomalies that relate to the size or increase of volume, may be either greater or less than in a state of health. Mass and volume are not necessarily both increased or diminished at one and the same time; it sometimes happens that they are met with in an inverse ratio in the same instance; an increase is much more frequent than a decrease in volume. In the heart, veins, and lymphatics, enlargement is generally complicated with a change of texture. An increase of substance of the heart accompanies this change; whilst in the vessels there is merely simple dilatation connected with it. Again, in the arteries, there is not only a change of texture, but a partial rupture also.

The morbid alterations of the heart do not come within the range of the present inquiry; and we begin, therefore, with the description of that morbid dilatation of arteries called aneurism. Nevertheless, under this name, several conditions of an artery, totally different from each other, are included; for example:—1st. The total or partial dilatation of their circumference; and 2d. the total or partial destruction of their continuity: the first being called the true aneurism; the second, the false or mixed aneurism. A true aneurism is commonly understood to be the simple dilatation of an artery in its whole circumference; but this is a very rare occurrence, and hardly ever takes place. It is more usual to meet with a slight dilatation, accompanied with a considerable alteration in the internal membrane of the artery,—such, for example, as either inflammation or the formation of cartilage, or fragility of that tunic; and, at a later period, when the dilatation has become more considerable, there is a rupture of the internal and fibrous membranes, and an effusion into the cellular texture, which is always more or less perceptible. The cellular membrane must have already contracted adhesions with the internal coat of the artery, either in consequence of the diseased condition of the latter, or from the effect of compression; so that, whether the rupture take place on a sudden or gradually, an effusion that shall be immediately fatal cannot take place. This is the reason that an artery is not found uniformly distended in an aneurism of a large size; but the sac produced by the cellular membrane is met with applied to its surface, and commonly united to it by a narrow neck. This sac contracts adhesions to the neighbouring parts, but the pressure of the blood destroys it little by little, or sometimes suddenly. When the portion destroyed corresponds to a bone, nothing escapes; but, when it is in contact with some organ which is not itself so protected, the blood escapes either slowly or rapidly, and death ensues. Such is the ordinary termination of the true aneurism.

It very seldom happens, when the tumor is left to itself, that inflammation, arising accidentally, obliterates the diseased portion of the vessel; or the circulation becomes re-established by the dilatation of the collateral branches.

This description proves that the greater number of aneurisms, usually called true, are really mixed; since there is a rupture of the internal tunic, together with dilatation of the outer coat.

Mere dilatation of the arteries takes place only in those coats which enlarge a good deal; because, under those circumstances, the artery participates in the increase of nutrition afforded. It does not unfrequently happen that the arterial system partakes of this morbid alteration for a considerable portion of its extent. The same person sometimes affords examples of a great number of aneurisms upon different parts of the body, and in different arteries of the same part; but such a predisposition is not absolutely necessary for the production of the disease, and, whether there be fragility of the internal tunic or not, the influence of external and mechanical excitement is sufficient to produce this condition.

The false, or *spurious*, aneurism hardly merits the name of this formidable disease, since it consists merely of a solution of continuity in the vessel, without any dilatation of its coats: it is commonly produced by some external injury, very often from bleeding, and therefore it is more commonly met with at the bend of the arm, in the brachial artery. It is also denominated circumscribed or diffused, according to the space it occupies: in the latter instance, the blood is diffused among the muscles of the arm; in the former, it is confined to a small space.

The *mixed* aneurism, according to the definition given by surgical writers, is that in which a laceration of some arterial tunics is met with, together with a simple distention of others: that is, there may be a rupture of the external coats with dilatation of the internal, or *vice versa*. This latter being the usual state of the parts, is what has usually been called a true aneurism.

But the former diseased condition must be of very rare occurrence; since neither the removal of the external nor the middle coat of an artery is productive of any dilatation of the vessel, even when the neighbouring parts afford it no support or protection.

Besides these diseased states, which only relate to the arteries themselves, it may happen that the wound of an artery may be accompanied by that of a vein: if these wounds are so situated that they open one into the other, a *varicose* aneurism is the consequence, which is sometimes, though erroneously, called a *mixed* one. This accident is, however, uncommon, and merely fortuitous in those aneurisms arising from an internal cause, usually regarded as true aneurisms. It may, nevertheless, happen that the sac may in that case open into a neighbouring vein, to which it might have previously adhered, the parietes of which it has at length destroyed at the point of contact. But it is much more common in those cases where an external injury produces the common false aneurism, and where the vein has been divided through and through into the cavity of the accompanying artery. The blood, in this case, flows from the artery into the vein, and into the surrounding cellular tissue; but the vicinity of the two vessels, the facility with which the blood passes from the artery into the vein, and the influence of compression, cause the corresponding orifices of the vessels, as well as the external wound, to close; and nothing remains but an unusual communication between the artery and the vein.

Dilatation of the veins, or of the lymphatics, is never accompanied, or very rarely, with disease of their membranes; for they are naturally more dilatable than the arteries. In general, also, they are caused merely by mechanical obstruction to the free passage of the contained fluids. Nevertheless, it sometimes happens that ruptures ensue, when the dilatation has gone to too great an extent. The partial dilatation of the lymphatics, which takes place between two pair of valves, and which are accompanied by the obliteration of the vessels, may give rise to a species of hydatid; which, however, is very rarely met with in the veins from this cause. Diseased dilatation of the vessels usually affects but one of these systems, but it is not very uncommon to find all three acted upon at the same time: it is, then, the capillaries that first take on the diseased action. The disease appears under the form of a red, pulsatile tumor, often subject to bleeding, composed of an intricate net-work of vessels, frequently met with in the subcutaneous cellular tissue, and which has been called aneurism by anastomosis.

The opposite condition of the vascular system,—that is, an unusual narrowness and diminutiveness,—is much less common than the preceding class of diseases. It is very uncommon to meet with it in more than in one species of vessels, and the narrowness of that one is often accompanied by an unnatural enlargement of another. This condition may be only a vice of conformation, or it may depend upon a morbid alteration of texture. To this circumstance must be attributed, more especially, the closing of the canal by the extravasation of a fibrous matter at the termination of inflammation; but most commonly this effusion does not merely narrow the vessel,—it obliterates it altogether. Vessels always diminish by degrees, become weaker, and are obliterated when blood no longer flows into them; whether this state belongs to the regular progress of organisation, as is the case in the obliteration of the umbilical arteries and vein; or whether it arises from some accidental cause.

With regard to the anomalies of situation and configuration of the vascular system, it is sufficient to observe, that the majority of these are congenital, and they are all reducible to two classes: the first comprehends those which produce only an irregularity in the circulation, as their consequenee; whilst the second embrace all those which form an unusual communication between the arterial and venous systems, and are the cause of that diseased appearance of the skin, known by the name of the blue disease.

The study of the changes that take place after the wounding of a vessel, especially either an artery or vein, is of the greatest importance. Total or partial solutions of continuity, punctures, wounds made with cutting instruments, either longitudinal or transverse, together with the phenomena produced by the ligature of a vessel, must all be examined.

The most ordinary cure of the wound in an artery, is by the obliteration of the vessel by the effect of inflammation, which is often established without a solution of continuity,—as when a ligature has been passed round a vessel, and is removed after having been strongly tied; the effect of which is to divide the two internal coats, without injuring the external one. The former are reduced to the condition of a wound, and converted into secreting surfaces, which afterwards contract adhesions with each other. In some experiments, made purposely with a view of ascertaining this point, adhesion took place directly,—that is, at the termination of an hour from the application of the ligature; for the pulse could not be felt on the side operated upon, even after the ligature had been removed. When a ligature is only tied, and is immediately removed, the circulation does not suffer any interruption at the time; though sometimes, but not always, the obliteration is afterwards established; which proves that it is not the result of the coagulation of the blood, but of the inflammation and exudation *(of lymph).* The artery is not merely obliterated at the point on which the ligature, or the compression, has immediately acted; almost always its cavity is effaced from that spot to the first branch given off. In that portion it is converted into a narrow cord, and at length disappears entirely; whilst the collateral branches are dilated to a degree more or less considerable. This is the necessary result where the vessel has been transversely divided in its whole extent. But punctures or wounds, which only include a small portion of the circumference of arteries, may heal without any obliteration of the cavity, or indeed without any diminution of its diameter,—or, at least, without its suffering any considerable diminution. It is then possible to cure certain wounds of arteries by cicatrization, without obliteration, although that is frequently the result of the method employed to form a cicatrix.

In all wounds of vessels, the following circumstances take place:—First, blood escapes from the opening, which coagulates if the wound be small, and does not interrupt the circulation; the effusion takes place only at the external surface of the artery, but the coagulated portion forms a little projection towards the interior of the vessel, and soon contracts adhesions with the edges of the wound, and with those portions of the external part of the vessel which immediately surround it. If the vessel has been cut entirely across, it is retracted in length, as well as diminished in calibre, after the escape of the blood. The blood which surrounds it, as well as that which remains in its cavity, coagulates, from whence arises a temporary obliteration; but afterwards the internal membrane of the artery inflames, the whole internal circumference of the vessel adheres, and its cavity is entirely effaced.

The alterations of tissue in the vascular system are the following:—

1st. Inflammation and its consequences, which act upon all parts of this system, and which frequently, by the exudation following it, causes the obliteration even of the largest trunks, especially in the venous and lymphatic systems; for it attacks principally their internal membrane. Neither is it uncommon to find that inflammation produces, especially in the veins, a chain of abscesses that form in the course of the vessel, opening externally, and causing, as they heal, the obliteration of the vein.

2d. Ossification is by no means a rare occurrence in the vascular system. It depends, without doubt, upon a greater afflux of blood to the part than ordinary; but this state does not deserve the name of inflammation. The principal phenomena of ossification in the vascular system, are the following:—Ossification has its seat always in the internal membrane; the newly-formed osseous substance appears always under the form of plates, of a greater or less size, which line a greater or less portion of the circumference of the vessel. It is very common that the membrane, at these points of formation, is either dèstroyed wholly or in part. Ossifications are formed almost exclusively in that part of the vascular system supplied with red blood. They are more particularly found in the arteries, especially in the descending aorta, below the diaphragm, in the arteries of the lower limbs, and in the left ventricle of the heart. The venous system, and every part carrying black blood, affords very few examples of ossification; but they are not unusually met with in the lymphatic glands, even in subjects but little advanced in life. Frequently, indeed, these bodies appear to be entirely ossified, but, if examined with attention, there will always be found some portion of their substance, varying in size, which has not undergone any alteration. Ossifications are more frequently met with in man than in the female: they are commonly the disease of an advanced period of life; nevertheless, they must always be considered as a diseased state of the vessels, and not the mere effect of old age,—since, although in Europe it is very common to meet with ossifications in old men, yet there are many very aged people who have no trace whatever of such formations; and they are very uncommon in certain countries,—in the West Indies, for example. Besides, they occasionally make their appearance in the young, in the pulmonary artery, as well as in the vessels carrying red blood. Allied to this condition of the arterial system, fragility of these vessels may be mentioned, which, although it may exist alone, almost always accompanies the development of the ossific formation.

Of all the portions of the vascular system, the lymphatic glands are the only parts subject to be converted into formations entirely diseased. These are sometimes primitive,—as, for example, in scrofula, where the lymphatic glands are found to enlarge, and terminate by becoming converted, either totally or partially, into masses of a whitish substance, greater or less in extent, which has at first a considerable degree of hardness, but changes afterwards into a grumous and whitish pus. These morbid alterations may also ensue in consequence of infection, when a contagious principle, absorbed in a part previously diseased, reaches a lymphatic gland, which reacts powerfully upon it, because the function of these glands consists in assimilating substances foreign to them; from thence arise inflammation, tumefaction, and suppuration of these organs, and their conversion into diseased tissues, analogous to those developed in the parts with which they had communication by means of the lymphatic vessels, and may be observed in various kinds of ulcers, in cancer, &c.

In our next Number we shall take up the diseases of another structure.

[To be continued.]

### MEDICAL AND PHYSICAL INTELLIGENCE.

